# Digital Sublimation Printing on Knitted Polyamide 6.6 Fabric Treated with Non-Thermal Plasma

**DOI:** 10.3390/polym13121969

**Published:** 2021-06-15

**Authors:** Marcia Cristina Silva, Gilberto Petraconi, Ricardo Rodrigues Ramos Cecci, Adriano Alves Passos, Wanderson Ferraz do Valle, Bruno Braite, Sérgio Ricardo Lourenço, Fernando Gasi

**Affiliations:** 1 Center for Engineering, Modeling and Applied Social Sciences, Federal University of ABC (UFABC), Santo André, São Paulo 09210-580, Brazil; bruno.braite@ufabc.edu.br (B.B.); sergio.lourenco@ufabc.edu.br (S.R.L.); fernando.gasi@ufabc.edu.br (F.G.); 2 Technological Institute of Aeronautics, ITA, São Jose dos Campos, São Paulo 12228-900, Brazil; petra@ita.br; 3 SENAI Innovation Institute for Biosynthetic and Fibres, SENAI CETIQT, Rio de Janeiro 20961-020, Brazil; rrcecci@cetiqt.senai.br (R.R.R.C.); apassos@cetiqt.senai.br (A.A.P.); wfvalle@cetiqt.senai.br (W.F.d.V.)

**Keywords:** plasma, sublimation, PA6.6

## Abstract

The garment industry demands stamping processes that are increasingly more agile and less damaging to the environment. In this scenario, digital printing, with the sublimation transfer printing technique, presents itself as a viable option for synthetic textile substrates. Among the synthetic fibres, polyamide (P.A.) fibres stand out, as they are light, soft, durable, and boast moderate sweat absorption; however, before sublimation, superficial treatment is necessary in order to present good results such as withstanding washing and maintaining colour intensity. This study addresses the surface modification of the PA6.6 textile substrate by activating non-thermal plasma at atmospheric pressure to receive dye through the sublimation method with dispersed dye. The knitted PA6.6 fabric surface treatment was performed with plasma application at atmospheric pressure using air in the Plasmatreater AS400 equipment. The sublimation transfer effects were evaluated by wash fastness and colourimetric tests. To assess the wettability effect of the control and treated samples, a contact angle test was carried out on PA6.6 samples. Fourier transform infrared spectroscopy (FTIR) proved the changes in chemical functional groups in the fibres. The results showed a decrease in the contact angle of the textile surface, 4–5 grayscale results for colour change and transfer for washing, and an increase in colour strength. In the FTIR tests, there is an increase in the transmittance value of aromatic, carboxylic groups (C=O, 580 cm^−1^), amides (N=H, 1630 cm^−1^), and methyl groups (CH 1369 to 1463 cm^−1^) as well as the presence of new functional groups in the 3064 cm^−1^ and 2860 cm^−1^ bands. These conditions allowed sublimation in the knitted PA6.6 fabric and showed increased colour strength and good wash fastness.

## 1. Introduction

The clothing industry demands faster printing processes and on minor scales to meet and keep up with fashion designs. Another major challenge for the textile industry is the search for a less environmentally damaging process—for example, the conventional dyeing process uses a lot of energy and a large volume of water and also has the need for effluent treatment [1,2]. Digital printing offers solutions for various textile materials. Among other factors, the choice of the process and the type of dye depend on the textile material fibres, the end use, and the possibility of pre-treating the textile substrate [3].

The process begins with the creation of the image and its printing on special paper with sublimated ink. The transfer of the image to the textile substrate is done using a table press or calendaring rolls, applying pressure and heat for a determined time. Direct printing on the fabric can also be done, but the dye is fixed using steam [4].

Dispersed dye is used in sublimation inks, this dye does not require a fixing agent; with the increase in temperature, it sublimates; that means that it changes from the solid to the gaseous state without going through the liquid form [4]. As the temperature rises, the textile fibres open spaces where the sublimated dye enters. As the temperature decreases, the fibres close and hold the dye in its solid state, providing the textile substrate with good wash and light fastness [5].

The number of synthetic fibres produced in the world exceeds the production of natural origin fibres. The polyamide fibre 6.6 (PA6.6) stands out, as it is a light, soft, durable fibre with moderate sweat absorption. Such conditions allow the structure of the knitted fabric to produce lingerie, sportswear, and socks. PA6.6 is a synthetic chemical polymeric fibre known as nylon, with excellent mechanical properties, including high tensile strength, high flexibility, and good chemical resistance, among others. The flexibility of the aliphatic segments in the amorphous regions of the polyamide is also responsible for its high tenacity [6,7]. They have excellent durability due to the low friction coefficient (self-lubricating), high melting temperature, and glass transition temperature, resulting in good mechanical properties at elevated temperatures [8,9].

PA6.6 is the polymer in which the structural units are linked by amide groupings (-CONH-). PA6.6 is obtained in linear polycondensation hexamethylenediamine (H2N (CH2) 6NH2). In polyamide 6.6, the first 6 represents the number of carbons in the diacid, and the second 6 represents the number of carbons in the diamine. It is important to define the concept of polymerisation per step, which occurs through the reaction between two different functional groups, for example, –O.H. and –COOH or –NH2; R-NCO and –O.H. or –NH2; where O.H. = hydroxyl; COOH = carboxyl; NH2 = amine. Then, each stage of the polymerisation of polyamide 6.6 is verified: nH2N (CH2) 6NH2 + nHO2C (CH2); H [NH―(CH2) 6―NHCO―(CH2) 4―CO] n OH + (2n − 1) H2O. Polymerisation proceeds with approximately 80% to 90% conversion [10,11].

The dyeing of P.A. fibres is done mainly with acid dyes. It connects the amino-terminal content groups of the fibres and the content of sulfonic groups of the dye in a reaction that occurs in an organic acid medium, with pH between 4.5 and 5.5. As the chemical bonds do not present the necessary conditions for dyeing, the dye sublimation technique dispersed in knitted PA6.6 fabrics has low washing strength [12].

Reactive dyes are also used in dyeing polyamide, as they have an electrophilic group capable of forming covalent bonds with amine groups in polyamides. The main groups of reactive dyes contain the azo and anthraquinone function; chromophoric groups, chlorotriazine, and sulfatoethylsulfonyl groups. Lightfastness results are similar to those obtained with acid dye; however, the colour depth is limited [13].

The process of dyeing PA6.6 fibres with dispersed dye obtains low wash fastness. Due to the interaction of the amine groups with the chromophores, many of the PA6.6 substrate’s shades are changed, and the colours are generally vivid, except for red shades [13].

The practical application of a textile material depends, among others, on technical characteristics, surface properties such as adhesion, gloss, permeability, dyeing, printing capacity, and surface cleaning, without changing the fibres’ bulk properties. Similar to dyeing, many treatments to modify the surface of the textile material are carried out in a humid environment and with chemicals that, after use, become industrial effluents, using large amounts of water and energy and placing workers in unhealthy environments [14].

With the modernisation of textile processes and a focus on sustainability, plasma technology has been used in various applications for textile surfaces treatment, and different surface modifications can be obtained with the selection of gas and the plasma’s process conditions [15], such as the use of plasma for mechanical changes and tactile performance in mixed wool/cashmere fabrics [16], the modification of the surface to improve the hydrophobicity of the textile substrate and make it more attractive for self-cleaning and waterproof product development [17], and the inhibition of the growth of microorganisms and the biodeterioration of natural fibre fabrics [18].

The process of modifying polymers’ characteristics and properties, and changing the chemistry on the surface is called plasma functionalisation. It presents itself as an advantageous alternative to wet chemical treatments when environmental and safety aspects are considered [19].

The physical principle of plasma technology is the change of state. A matter fed with energy passes from the solid state to the liquid and from the liquid to the gaseous. When the gaseous matter is provided with energy, ionisation occurs, entering the plasma state, where electrons are released from atoms or molecules [20]. The electrons in the plasma acquire energy in the range of 0.1–10 eV. The ions and molecules reach energy in the range of 0.025 eV, without presenting a condition of thermodynamic equilibrium, without which neutral ions and molecules disintegrate [21,22].

Most textile materials are heat-sensitive polymers, so the type of plasma used in textile processes is non-thermal plasma or cold plasma and can be applied at atmospheric pressure or low pressure. High-energy electrons and low-energy molecular species can initiate reactions in the plasma volume without excess heat that causes degradation of the textile substrate [23,24].

The types of plasma that can be used on textile substrates and atmospheric pressure are plasma jet (PJ), glow discharge (GD), corona discharge (CD), and dielectric barrier discharge [8]. The PJ operated at atmospheric pressure is a recent technique for modifying the surface of textile substrates that enables smooth and highly effective application at room temperature. The advantage of DBD is that it can be applied to any geometric surface [12].

Active species and energetic electrons present in plasma can break chain bonds, generating free radicals on the surface. As a result of this, there is the possibility of cross-linking on the surface or creation of functional groups such as carboxylic acid groups, hydroxyl groups, and amine groups, due to the interaction of free radicals with plasma species, such as ozone or when it comes into contact with ambient air [19,21].

Many researchers have researched the application of non-thermal plasma and atmospheric pressure, for example, surface modification of polyester textile substrates to receive silicone nano emulsion [23] and surface modification of polyamide nets to receive antifouling treatment for use in fish farming [24].

This work addresses the surface modification of the PA6.6 substrate with the non-thermal plasma treatment under atmospheric pressure in receiving dye through the sublimation method using dispersed dye and presents values of high durability to washing and colour strength. For the textile industry, this study is critical because it breaks the paradigm that it is impossible to make sublimation print transfer on PA6.6 substrate, obtaining vivid colours and with a solid wash.

## 2. Materials and Methods

### 2.1. Materials

#### 2.1.1. Knitted Fabric

Plasma treatment was applied on a knitted fabric made with 1x 80/68 PA6.6 yarn with 20 denier elastane, made on a circular loom with 38 needles per inch with a weight of 180 g/m^2^. The same fabric, without plasma treatment, was used as a reference to evaluate the results.

#### 2.1.2. Sublimation Transfer Printing

The preparation of sublimation transfer printing was done on the Printer: Epson SureColor F9200, loaded with Epson UltraChrome^®^ DS Sublimation ink on Epson Sublimation 90 g/m^2^ paper.

### 2.2. Methods

#### 2.2.1. Preparation of Samples and Plasma Treatment

The knitted PA6.6 fabric was cut into 25 × 60 cm specimens and sent to the plasma application.

**Figure 1:**
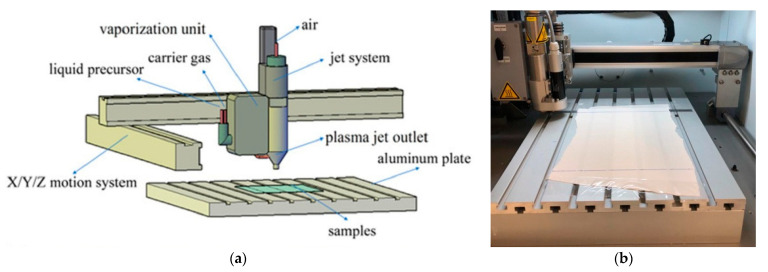
(**a**) Schematic drawing of the experimental apparatus [25] and (**b**) Sample of PA 6.6 textile substrate submitted to plasma treatment.

Plasma treatment was performed using the Model AS400 Atmospheric Pressure Plasma System manufactured by Plasma Treat GmbH (Steinhagen, Germany), which was installed at the Beneficiation Laboratory of SENAI CETIQT (Figure 1).

The workspace for the discharge of luminescent plasma has the dimensions of 33 cm by 12.5 cm, and the plasma system is powered by an R.F. energy source (19 kHz).

Atmospheric air was used to generate the plasma. The substrates were fixed on an aluminium plate, and the plasma jet was controlled by an X/Y/Z movement system. The plasma voltage was adjusted to 300 V, the current was adjusted to 0.3 A, and the power was adjusted to 480 W. The distance between the sample and the nozzle was kept constant at 12.5 mm. The jet travel speed and the number of passages for each sample are described in Table 1. All experiments were carried out under controlled conditions.

#### 2.2.2. Sublimation

The material was subjected to sublimation in a 38 cm × 38 cm flat press with a LIVE brand drawer, with an alligator opening, at a temperature of 180° and a time of 15 s. The colours applied were red, yellow, and blue.

#### 2.2.3. Contact Angle

The sessile drop method, which measures the contact angle (θ) of a drop of water on the material surface, was used to characterise the wettability properties of the knitted 6.6 polyamides fabrics before and after plasma treatment. A surface analyser (Drop shape analyser) DSA-100, Kruëss Co, Hamburg, Germany was used for the measurement. The samples were placed on the base of the analyser, and a drop of water was introduced on the surface using a micro-syringe. The contact angles at steady state were measured using a 1 µL drop of deionised water. At least three different measurements were performed at room temperature and evaluated with the aid of the Advance software. All samples were analysed with the same light settings and adjustment algorithms. The value of the contact angle, θ, indicates whether the surface is hydrophilic or hydrophobic: θ < 90° corresponds to a hydrophilic surface, while θ > 90° implies a hydrophobic surface [26].

#### 2.2.4. Wash Fastness

According to the technical standard ABNT NBR ISO 105-C 06: 2006, the sublimated material was subjected to a wash fastness assessment to determine the degree of fastness of a given colour and the gaining of the dye from the processed fabrics.

The greyscale was used to assess durability, according to technical standards ABNT NBR ISO 105-A02: 2006: grayscale to determine colour change, ABNT NBR ISO 105-A03: 2006: Greyscale to assess colour transfer, which varies from 1 to 5 with 5 being the maximum score, that is, when there is no colour change [27,28,29].

#### 2.2.5. Colour Measurement and Colour Strength

For the colourimetry tests, eight simple measurements were made in the included specular geometry (SCI). The average of the eight sets of reflectance values was used. The equipment used for measuring was a spectrophotometer of the brand Minolta, model CM-3720d. The reflectance values were used to construct the colour representation space in the Ciel standard (L* a* b). The comparison between the samples is obtained by the Euclidean Distance (ΔE) in that colour space. Colouristic strength was calculated using the Kubelka–Munk theory [30].

#### 2.2.6. FTIR (Fourier Transform Infrared Spectroscopy)

The samples were analysed using the spectroscopy technique in the mid-infrared region (700 to 4000 cm^−1^) to obtain quantitative and qualitative information on the material under analysis. This study will be applied to evaluate the number of chemical components that contribute to the molecular formation in the fibres under investigation. A BRIRKER VERTEX 80 FTIR device was used, with a resolution of 4 cm^−1^, 16 scans, a sample scan time of 16 s, a background scan time of 16 s, a measurement time of 1 s, a range of 4500 cm^−1^–370 cm ^−1^. Resulting spectrum: transmittance, Accessories: 4225/Q Platinum ATR, Multiple Crystals CRY: Diamond, Beamsplitter: KBrO.

#### 2.2.7. Scanning Electron Microscopy (SEM)

SEM analyses performed using a Thermo Scientific microscope (Waltham, MA, USA) model Prisma E, from the SENAI CETIQT Characterization Laboratory, equipped with an energy-dispersive X-ray spectrometer (EDS). The equipment was operated with an electron acceleration voltage of 20 kV in backscattering and secondary electrons. Preparation samples by gold deposition (sputtering) using an SC7620 Mini Sputter Coater/Glow Discharge System by Quorum. A vacuum with an ultra-pure argon atmosphere was used, applying a current of 15 mA for 60 s and a gold target.

## 3. Results

### 3.1. Contact Angle—θ

One of the methods for confirming the outer chemical structure is surface wettability verification. It is possible to identify hydrophobic and hydrophilic functional groups in the surface layer of the polymer [31]. The measurement of the static contact angle of the water drop (SWCA) assesses the effect of the jet plasma application speed on the surface wettability.

PA6.6 surface wettability substrate after plasma treatment was carried out with the SWCA measurement in samples treated with plasma varying the number of passages from three to four in the control sample. All analyses were performed immediately after treatment at three dry points in the samples.

**Figure 2:**
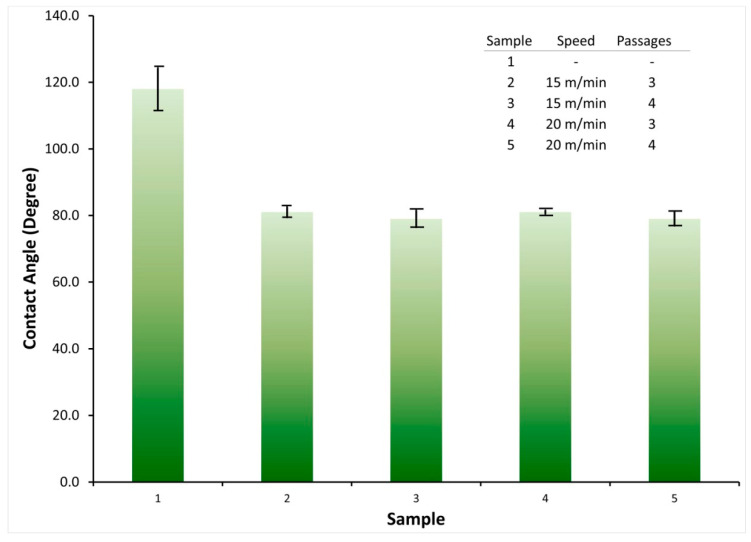
Measurement of the contact angle for PA6.6 textile substrate samples submitted to surface treatment with atmospheric plasma and control sample. Application speed 15 m/min and speed 20 m/min. Contact angle at steady state was measured after placing the drop of deionised water on the fabric surface (t = 0) and immediately after plasma application.

Figure 2 shows the SWCA data collected during the first seconds of the water drop contact with the PA6.6 surface at the plasma speed conditions of 15 and 20 m/min.

The initial value of the contact angle of the control sample was (118.0 ± 7.0)°, which demonstrates the hydrophobic nature of the substrate tissue of PA6.6. These results show that the surface before the treatment was classified as not wettable (θ is greater than 90°), and the contact angle θ starts to reduce with each passage of the atmospheric plasma, becoming wettable (θ less than 90°) [26].

For the substrates treated with a speed of 15 m/min, they presented values of (81.0 ± 1.5)° and (81.0 ± 0.6)° for three and four passes, respectively. When the speed is increasing to 20 m/min, the results for SWCA were (78.0 ± 3.0)° and (78.0 ± 2.5)° for three and four passes, respectively. These results indicate the change in the surface of the samples from hydrophobic to hydrophilic. This can be explained by the concentration of polar functional groups such as hydroxyl (OH), carboxyl (COOH), and amino (NH_2_), as these groups become soluble in a polar solvent, water, which is a situation that allows water to spread on the surface [19]. The verification of the functional groups’ presence will be in the FTIR analysis.

### 3.2. Test for Colourfastness

The colour change and the transfer in the washing of the sublimated fabric were classified as good or very good (4–5; 5). The colour change was between 4 and 4–5. It is possible to observe in Table 2 that there was no significant difference in the transfer of samples between cotton treated with plasma and cotton without treatment, highlighting the improvement in the transfer of the colour red.

### 3.3. Colourimetry and Colouristic Strength

It was possible to observe that the colours of the fabrics submitted to the plasma treatment are more intense than the untreated fabric (Table 3). The luminosity values (L*) are lower in the materials treated for all colours, except for sample 4, blue (with plasma application at 20 m/min and three passages).

It is possible to observe that the coordinates a* and b* increase in the colour red in the treated samples. In the colours blue and yellow, the coordinates oscillate higher or lower than the coordinates of the untreated sample.

**Figure 3:**
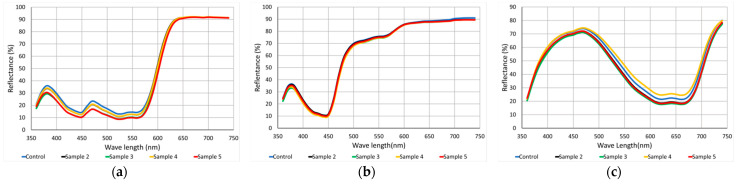
Reflectance × wavelength of colour (**a**) red; (**b**) yellow; (**c**) blue.

It is possible to observe in Figure 3 that almost all the samples with plasma applied increased the colouristic strength, except for sample 4 of the colour blue and sample 2 of the colour yellow. The control sample before and after washing was considered the standard sample. The colour red achieved good results (159.9%) of colouristic strength, reaching 60% more power than the control sample. The blue colour achieved 134.5% of colouristic strength, showing the efficacy of the plasma treatment in the sublimation of the PA6.6 textile substrate.

However, after the samples were washed, the colour strength dropped dramatically. The strength reached 35% more than the control sample for the colour blue, and even after washing, the samples still showed considerable colour strength. The colour yellow showed the lowest values of colouristic power, being 20% stronger than the sample without treatment, and there was no significant change after washing. This variation may be associated with the instability of atmospheric plasma and corrected with the variation of the applied energy.

### 3.4. FTIR Analysis

**Figure 4:**
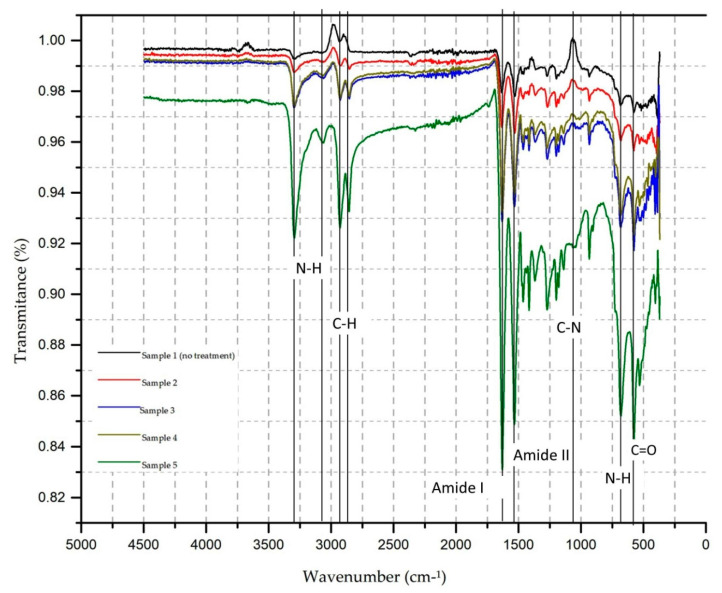
FTIR spectra of PA6.6 textile substrate: 1—control sample (black line); samples with plasma treatment 15 m/min three passages (2—red line), four passages (3—blue line); samples with plasma treatment 20 m/min three passages (4—brown line) and four passages (5—green line).

To assess changes in the functional polyamide 6.6 groups before and after surface treatment with atmospheric plasma, Fourier transforms infrared spectrometry (FTIR) performed assays in the medium infrared. The resulting spectrum is shown in Figure 4.

When a molecule is illuminated by infrared light, the absorbed light became a molecular vibration. The infrared spectrum represents the transmittance value, the energy ratio that strikes the sample, and the energy transmitted at each frequency value or number of waves. The vibrations can be of the type of axial deformation and angular deformation.

Sample 1—PA6.6 control sample has the bands inherent to the material observed at 3290 cm^−1^ (free NH asymmetric axial stretch), 2927 cm^−1^ and 2850 cm^−1^ (CH stretch in CH2), 1633 cm^−1^ (amide I), 1538 cm^−1^ (δ NH + υ group of CN—amide II), 1371 cm^−1^ (CN stretch with NH deformation), 1262 cm^−1^ (axial deformation group CN), 1199 cm^−1^ (carbonyl vibration mode in crystalline phase), and the bands that can be used to characterise the amide group in polyamides are 690 cm^−1^ group (NH), 580 cm^−1^ (group C=O) [32].

In Figure 4, it is possible to verify that plasma-treated samples (2, 3, 4, and 5) demonstrate an increase in the transmittance values of the groups corresponding to C=O (580 cm^−1^) and NH (690 cm^−1^) in the region of amide IV, CH (1396 to 1463 cm^−1^), (1532 cm^−1^) in the region of amine II, and NH (1630 cm^−1^) in the region of amide I [33]. These changes may be associated with changes in the microenvironment and oxygen to the treated surface and contribute to the fabric dyeing affinity [22,32]. The increase in the transmittance values was also verified for the NH groups. There is symmetric and asymmetric CH stretching in the bands of 3290 and 2860 cm^−1^, which can be related to the formation of low molecular weight etched materials resulting from the treatment of the textile substrate and corroborate the established method to produce hydrophilic surfaces in polymers by introducing new polar groups [19].

**Figure 5:**
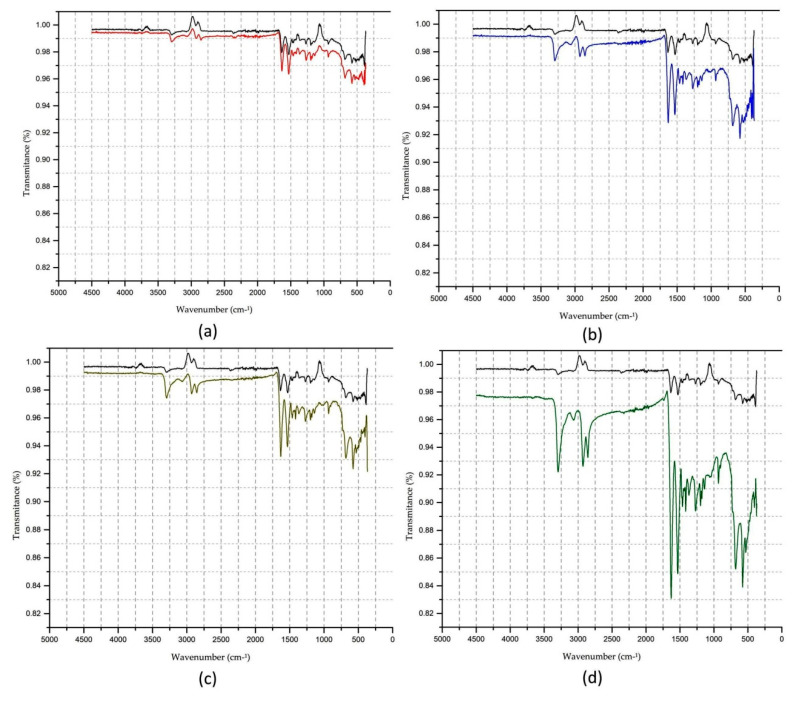
Comparison of FTIR spectra of PA6.6 textile substrate: (**a**) Sample 1—control, no plasma treatment × sample 2—plasma-treated 15 m/min, three passages; (**b**) Sample 1—control, no plasma treatment × sample 3—plasma-treated 15 m/min, four passages; (**c**) Sample 1—control, no plasma treatment × sample 4—plasma-treated 20 m/min, three passages; (**d**) Sample 1—control, no plasma treatment × sample 5—Plasma-treated 20 m/min, four passages.

**Figure 6:**
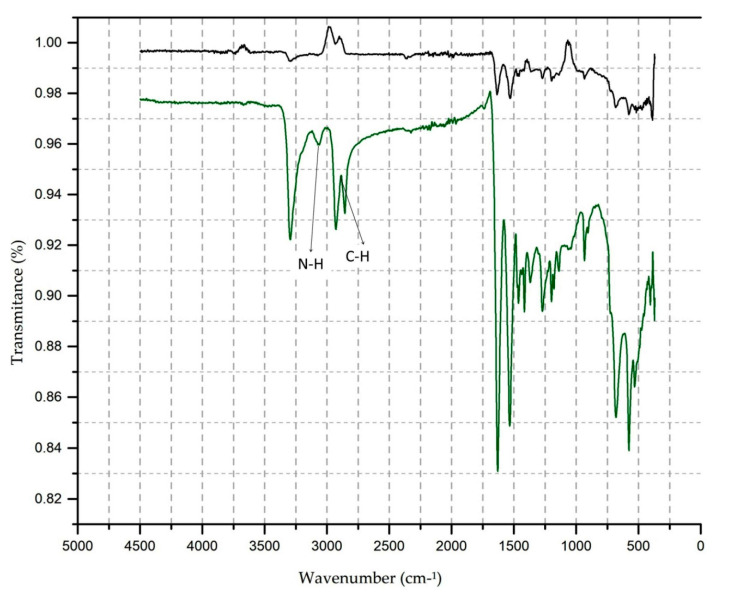
Comparison of FTIR spectra of PA6.6 textile substrate: Sample 1—control, no plasma treatment × sample 5—Plasma-treated 20 m/min, four passages.

In Figure 5b–d, it is possible to observe that new functional groups appear, which are associated with the vibration of symmetrical stretching in the CH region in the 2860 cm^−1^ band and the NH region in the 3064 cm^−1^ bands. To illustrate more clearly the position of the new functional groups, the comparison between sample 1 and sample 5 is shown in Figure 6.

In Figure 6, it is possible to observe the location of the new functional groups in a magnified way. For each adjustment in the test parameters and after plasma treatment, it is possible to observe the appearance of new functional groups and increase transmittance until reaching sample 5 (20 m/min, four passages), with the most striking difference between the control sample and the treated sample.

Therefore, the electron energy induced by applying atmospheric plasma on the knitted PA6.6 fabric’s surface causes the polymer bonds to break with energy less than 10 eV of the C-N bond, precisely because it is the weakest chain. After being broken and in contact with the reactive species created by the plasma, it can generate a low molecular weight molecule containing carboxylic and amino groups.

The changes in the properties of the textile surface produced by plasma treatment are strongly related to the differences in the transmittance values, which correspond to the CH, NH, and CN groups.

This condition provides a greater affinity for dyeing, allowing for the realisation of sublimation transfer printing with excellent fastness characteristics to washing and colour intensity, including increasing the three colours’ colour strength.

### 3.5. Scanning Electron Microscopy (SEM)

Scanning electron microscopy (SEM) images were obtained. In Figure 5, it is possible to observe the PA6.6 control sample’s surface and compare it with the samples submitted to plasma treatment.

**Figure 7:**
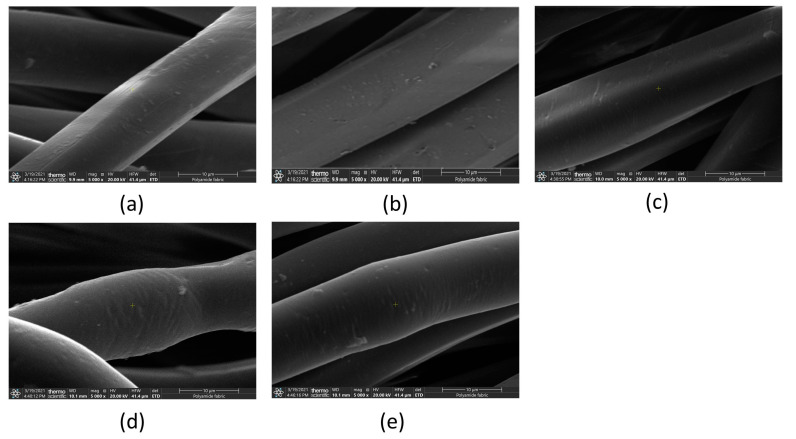
SEM for PA6.6 textile substrate. Amplification 5000×. (**a**) 1—Control samples subjected to atmospheric plasma surface treatment; (**b**) 2—speed 15 m/min, three passages; (**c**) 3—speed 15 m/min, four passages; (**d**) 4—speed 20 m/min, three passages; (**e**) 5—speed of 20 m/min, four passages.

As previously demonstrated (in Section 3.1), the plasma modified the wettability of the polyamide fabric. For this reason, in this section, the SEM was used to verify the surface modifications in the samples. A comparison of the results shown in Figure 7 did not observe significant changes in surface roughness on amplification used.

## 4. Conclusions

The sublimation process consists of transferring the print paper to the textile substrate. It provides agility in the production and development of new products.

The contact between the paper and the substrate is intense, fast, and occurs at an average temperature of 180 °C. The textile fibre most used in this process is polyester (PES), as the results are characterised by high colour intensity and excellent wash fastness.

Polyamide 6.6 (PA6.6) fibre, on the other hand, has different characteristics from that of PES, as it provides greater thermal comfort and moisture absorption than PES (0.4% for PES and 4% for PA6.6).

This difference explains the interest in validating the sublimation process in PA6.6, which is an objective that had not been achieved until then.

Until today, the results obtained with this technique in PA6.6 did not reach the colour intensity demanded by the designs and high levels of fastness to washing. The results found in this study present an innovative solution, which is already in the process of obtaining a patent for textile dyeing processes in Brazil and in the world for sublimation transfer printing on the PA6.6 substrate with the use of dispersed dye in the sublimation ink.

The developed phases of the project will be described below, as well as the conclusions obtained.

1. Decrease in the contact angle of the static drop on the material surface. The increase in hydrophilicity on the surface of the textile substrate is essential so that there is a more significant dye absorption at the time of sublimation

From the results presented, it was possible to notice that the plasma treatment decreased the contact angle formed by the drop on the surface, which shows the increased hydrophilicity of the textile substrate with water.

The plasma treatment was able to modify the surface of the textile substrate, making it hydrophilic (greater absorption power). The initial value of the contact angle of the control sample was (118.0 ± 7.0)°. After treatment of the substrate with plasma, the results were as follows: for the substrates treated with a speed of 15 m/min, they presented values of (81.0 ± 1.5)° and (81.0 ± 0.6)° (speed 15 m/min, three and four passes, respectively). When the speed is increased to 20 m/min, the results for SWCA were (78.0 ± 3.0)° and (78.0 ± 2.5)° for three and four passes, respectively.

2. Colourimetry and colouristic strength

From the colourimetry analyses, it is noted that there was an apparent increase in the colour strength of the colours red, blue, and yellow, in the samples that were subjected to plasma treatment. The most significant gains were in the colour red applied in sample 5 with a colouristic force of 159.9% and the colour blue applied in sample 3 with a colouristic force of 134.6%. After washing, the colour strength of the colour red was considerably reduced, while those of the colours blue and yellow did not show any significant change.

3. Test for colourfastness

Concerning the washing strength, there were no significant differences between the control sample and the treated samples. This result validates the sublimation process on the PA6.6 substrate due to the high values of fastness to washing presented.

4. FTIR analysis

In the FTIR tests, there is an increase in the transmittance value of aromatic, carboxylic groups (C=O, 580 cm^−1^), amides (N=H, 1630 cm^−1^), and methyl groups (CH 1369 to 1463 cm^−1^), for the NH groups and symmetrical and asymmetric CH stretching in the bands of 3290, 3068, and 2860 cm^−1^.

The new functional groups are essential, and they are associated with the vibration of symmetrical stretching in the CH region in the 2860 cm^−1^ band and the NH region in the 3064 cm^−1^ bands.

As shown in Figure 6, the property changes of the textile surface produced by plasma treatment are strongly related to the differences in the transmittance values, which correspond to the CH, NH, amide, C=O, and CN groups and the presence of new functional groups associated with symmetrical stretching vibration in the CH region in the 2860 cm^−1^ band and the NH region in the 3064 cm^−1^ bands.

These changes (increased transmittance and new functional groups presence) allowed sublimation in the PA6.6 textile substrate and may explain the increase in colour strength and good wash fastness.

5. Scanning electron microscopy (SEM)

The images of the control sample and the samples treated with plasma, despite the change in the surface of the textile substrate, did not show significant changes in the roughness of the material.

## Figures and Tables

**Table 1:** Experiment’s parameters.

Sample	Speed	Passages
1 (Control)	-	-
2	15 m/min	3
3	15 m/min	4
4	20 m/min	3
5	20 m/min	4

**Table 2:** Colour fastness to wash.

Colour	Sample	Plasma Application		Colour Change	Colour Staining					
		Speed (m/min)	N		WO	PAC	PES	PA	CO	CA
Red	1	Control			5	5	4–5	4	4–5	3–4
	2	15	3	4–5	5	5	4–5	4	4–5	3–4
	3	15	4	4	4–5	5	4–5	4	4–5	3–4
	4	20	3	4	4–5	5	4–5	4	5	3
	5	20	4	4	4–5	5	4–5	4	5	3
Blue	1	Control			5	5	5	4–5	5	4–5
	2	15	3	4–5	5	5	5	4	5	4–5
	3	15	4	4–5	5	5	5	4	5	4
	4	20	3	4–5	5	5	5	4	5	4–5
	5	20	4	4–5	5	5	4–5	4	5	4–5
Yellow	1	Control			5	5	5	4–5	5	4–5
	2	15	3	4–5	5	5	5	4–5	5	4–5
	3	15	4	4–5	5	5	5	4–5	5	4–5
	4	20	3	4–5	5	5	5	4–5	5	4–5
	5	20	4	4–5	5	5	5	4–5	5	4–5

N—Passage quantity; CA—Acetate; CO—Cotton; PA—Polyamide; PES—Polyester; PAC—Acrylic; WO—Wool.

**Table 3:** Colour reading in CIELAB space for polyamide fabrics dyed by sublimation transfer printing, illuminant D65/10°.

Colour	Sample	D65/10°				
		L*	a*	b*	ΔE	Colour Strength (%)
Red	1 (Control)	58.89	44.93	15.85		
	2	57.58	48.44	18.05	1.81	119.5
	3	54.85	51.21	20.01	3.58	154.99
	4	57.44	47.9	17.6	1.54	121.96
	5	54.08	50.98	19.01	3.43	159.9
Blue	1 (Control)	72.29	−18.19	−26.15		
	2	70.02	−18.24	−28.57	1.59	124.26
	3	69.00	−18.09	−29.38	2.2	134.6
	4	74.24	−17.74	−23.26	1.72	80.95
	5	69.62	−17.97	−29.17	2	127.89
Yellow	1 (Control)	89.11	−8.14	55.00		
	2	88.98	−7.89	54.69	0.18	99.14
	3	88.50	−7.6	56.89	0.85	117.83
	4	88.48	−7.67	57.7	1.11	119.96
	5	88.76	−7.81	55.73	0.37	106.32

## Data Availability

The data presented in this study are available on request from the corresponding author.
